# Quality indicators of colonoscopy care: a qualitative study from the perspectives of colonoscopy participants and nurses

**DOI:** 10.1186/s12913-022-08466-5

**Published:** 2022-08-19

**Authors:** Wenwen Cai, Xingxing Zhang, Yanxia Luo, Minshan Ye, Yu Guo, Weiqing Ruan

**Affiliations:** 1 Huiqiao Medical Center, Nanfang Hospital, Southern Medical University, Guangzhou, Guangdong China; 2 School of Nursing, Southern Medical University, Guangzhou, Guangdong China; 3 Department of General Surgery, Nanfang Hospital, Southern Medical University, Guangzhou, China

**Keywords:** Colonoscopy, Nursing quality indicators, Colonoscopy participants, Nurses, Qualitative study

## Abstract

**Background:**

Quality of care in colonoscopy is closely related to colonoscopy participants and the nursing workforce in endoscopy-related settings. However, limited data are available on the evaluations and recommendations regarding quality indicators for nursing care by these two groups. Therefore, the aim of this study was to explore the standards and requirements of quality of care in colonoscopy from the perspectives of patients and nurses.

**Method:**

With a descriptive qualitative study, semi-structured interviews were conducted between November 2021 and January 2022 with colonoscopy participants (*P* = 11) and nursing workforce (*N* = 7) in the endoscopy unit in a tertiary hospital. The interviews were analyzed using a thematic analysis.

**Results:**

Nine major themes emerged according to the structure, process, and outcome care quality model: workforce structure, quality requirements, unit facilities, nursing tools, nursing quality control systems, dynamic assessment and intervention, pre-examination care, strengthening education, and colonoscopy outcomes.

**Conclusion:**

The indicator of quality of colonoscopy care should be used to assess and improve current practices to ensure a more direct and sustained impact of colonoscopy care. This study highlights the importance of nurse managers valuing the opinions and reflections of people involved in colonoscopy to improve the quality of colonoscopy care.

**Supplementary Information:**

The online version contains supplementary material available at 10.1186/s12913-022-08466-5.

## Background

Colorectal cancer (CRC) is the third most common cancer worldwide and the second leading cause of cancer-related deaths; the number of new CRC cases is approximately 1.9 million cases and the number of deaths is approximately 930,000 worldwide in 2020 [1]. With the global spread of CRC screening and surveillance, the number of colonoscopies, the gold standard for CRC screening, has steadily increased [2]. Quality colonoscopy decreases the risk of CRC incidence and mortality by 40% to 60% by reducing the adenoma miss rate (AMR) and interval CRC, while also enhancing the satisfaction of participants [3–5]. Clear quality indicators have been widely adopted [5]. Commonly used key metrics are designed to monitor endoscopist practices, including the ‘cecal intubation rate’, ‘adenoma detection rate’, ‘withdrawal time’, ‘complication rate’, and ‘surveillance intervals’ [6], which do not correlate strongly with the quality of colonoscopy care. Nurses play an important role as primary caregivers during colonoscopy, and the participation of experienced nurses in colonoscopy directly improves the detection rate of polyps and adenomas [7]. In order to assess the level of nursing activities for colonoscopy and to manage these activities quantitatively, a system nursing quality indicators is needed, which is objective, and highly nursing-specific [8, 9]. And indicator data can be collected at work. A qualitative survey of healthcare professionals concluded that improved communication and information sharing among practitioners could facilitate the colonoscopy process improvement [10]. The importance of professional nurses responding to the needs of each patient was also emphasized [11]. To ensure patient safety, a process framework for detecting and managing poor performance of care was established under the guidance of the Joint Advisory Group on Gastrointestinal Endoscopy (JAG) [12]. Nevertheless, the measurement scales for assessing nurses’ practice activities are quite limited. Additionally, with the advancement of patient-centered care (PCN), there is a need to increase patient participation in decision-making [13]. Based on this, in order to investigate the colonoscopy experience of patients, the production of the Global Rating Scale (GRS) for endoscopy was introduced in Scotland [14]. And patients report that inadequate bowel preparation is one of the biggest challenges, leading to increased length of stay and higher healthcare costs, so guidelines actively recommend updated minimum standards of bowel preparation aimed at increasing patient tolerance and improving the experience of the visit [15, 16]. It is evident that patients can play a more direct and continuous role in identifying, implementing, evaluating, and improving care.

Therefore, in order to enhance existing quality improvement strategies, additional research is essential to explore the suggestions of clinical nurses and participants for colonoscopy care. The overall objective of this study was to construct quality indicators of colonoscopy care based on the Donabedian structure, process, and outcome care quality model, which are divided into three categories: structure represents the meaning of equipment resources, human resources, and organizational structure; process refers to the actual nursing measures provided by the nursing workforce and the content of nursing activities received by patients; and outcome refers to the impact of nursing care on the health status of nursing clients, including nursing satisfaction, incidence of adverse events, and mortality [17].

## Objective

The specific objectives of this study were to to identify indicators for quality of care by (1) exploring patients’ perception of nursing-related intervention and by (2) exploring nurses’ perceptions of how to optimize the quality of care.

## Methods

### Design

This qualitative study used a phenomenological approach. The purpose of descriptive phenomenological research is to explore, analyze, and describe a near-real phenomenon, which is a non-complementary description of experience, rather than to generate a theory or explain it [18]. We declare that all methods were carried out in accordance with relevant guidelines and regulations. And the consolidated criteria for reporting qualitative research (COREQ; Supplementary File 1) were used to report the study [19].

### Participants and setting

The subjects of this study were recruited using a purposive sampling method. The inclusion criteria for the colonoscopy participants were as follows: (1) undergoing colonoscopy, (2) agreeing to share their experience, (3) age ≥ 18 years, regardless of gender, and (4) being able to read and express in Chinese and no mental abnormality. The exclusion criteria were as follows: (1) the interview was interrupted and (2) participants voluntarily withdrew or developed serious complications. Nurses who worked at the endoscopy unit for more than 5 years and were willing to participate were included in this study. Nurses whose interviews were interrupted or those who voluntarily withdrew were excluded from this study. Participants were invited to conduct the interview at the conscious moments after the examinations, and the schedules of nurses’ interviews were determined by themselves. Interviewee recruitment for this study was conducted concurrently with data analysis, and recruitment was terminated when data saturation was reached [20]. Eighteen face-to-face, one-on-one semi-structured interviews, ranging in duration from 12 to 37 min, were eventually conducted from November 2021 to January 2022 in a separate endoscopy unit at a tertiary hospital in Guangzhou, China.

### Data collection

Original interview guidelines were developed based on the objectives of this study and relevant references. After two pre-interviews, we improved the interview guidelines based on the comments of the interviewees, such as breaking down some questions, avoiding some medical jargon, and adjusting the interview protocol and focus (data from these two pre-interviewees were not included in the final outcome statistics). The formal outline, which included the interview informed consent form, interview guidelines, and general information about the interviewees, was finally reviewed by a senior and experienced expert in the endoscopy unit.

Ultimately, interviews were completed by an experienced female researcher who was not a member of the endoscopy unit, according to the interview guidelines listed in Table 1.. Interviewees were briefed on the purpose, format, and approximate time of the interview at the beginning, and the interviews were recorded with the interviewees’ approval. Opening paragraph was used to create a relaxed interview atmosphere. The researcher used appropriate silence, endorsement, and follow-up questions during the interviews and remained neutral, non-judgmental. The researcher used the interview guide as a reminder to make appropriate adjustments to the interview style according to the pace of the interview. Keywords and nonverbal expressions of the interviewees were consistently recorded. At the end of the interview, all interviewees were asked about personal data variables and assigned code names instead of their real names.

**Table 1.** Interview guidelines

Colonoscopy participant	Nurse
Could you please describe the experience of this colonoscopy?	Could you please tell me about your general awareness of quality indicators for colonoscopy?
Could you please tell me what impressed you most during your colonoscopy? Why?	Could you please tell me the difficulities you meet in your work? How were these difficulties resolved?
What makes you feel satisfied/unsatisfied? Were all your needs responded by the nurses?	Could you please tell me what key points need to be included in a colonoscopy care indicator system with reference to the structure, process and outcome models? Why?
Could you please tell me what else the nurse can do to resolve your inconvenience during the colonoscopy?	Is there anything else you would like to complete regarding the quality of colonoscopic care indicators?
From your personal perspective, what do you think a excellent colonoscopy nurse looks like? How can the quality of colonoscopic care be evaluated?	

### Data analysis

Data were processed using thematic analysis [21], and no data analysis software was used. To gain insight into the data, the text was transcribed verbatim by the interviewer who repeatedly listened to the recordings. Two researchers read the text data separately, performed an initial search to classify the data, and then used different codes to analyze and classify the text data. The data review continued until no new codes emerged. Ambiguities that could not be resolved between the two researchers were discussed with other team members to reach a consensus. The codes were then aggregated and analyzed by the structural, process, and outcome models, and all themes were decided by all members of the research team to ensure the reliability and validity of the analysis. Because the interview quotes used in the results of this paper needed to be translated from Chinese into English, the interview researcher consulted closely with English language professionals during the translation process to ensure equivalence of meaning.

### Ethical considerations

This study was submitted to and approved by the Ethics Committee of Nanfang Hospital, Southern Medical University, Guangzhou, China, prior to recruitment of interview subjects (NFEC-2021–378) and was also pre-registered with the China Clinical Trials Registry (registration number: ChiCTR2100054110). Informed consent was obtained from all interviewees prior to each interview. In addition, colonoscopy participants were assured that their right to receive other treatments and their nurse-patient relationship would not be affected. To ensure the privacy of each participant, all study data were displayed anonymously.

## Results

**Fig. 1:**
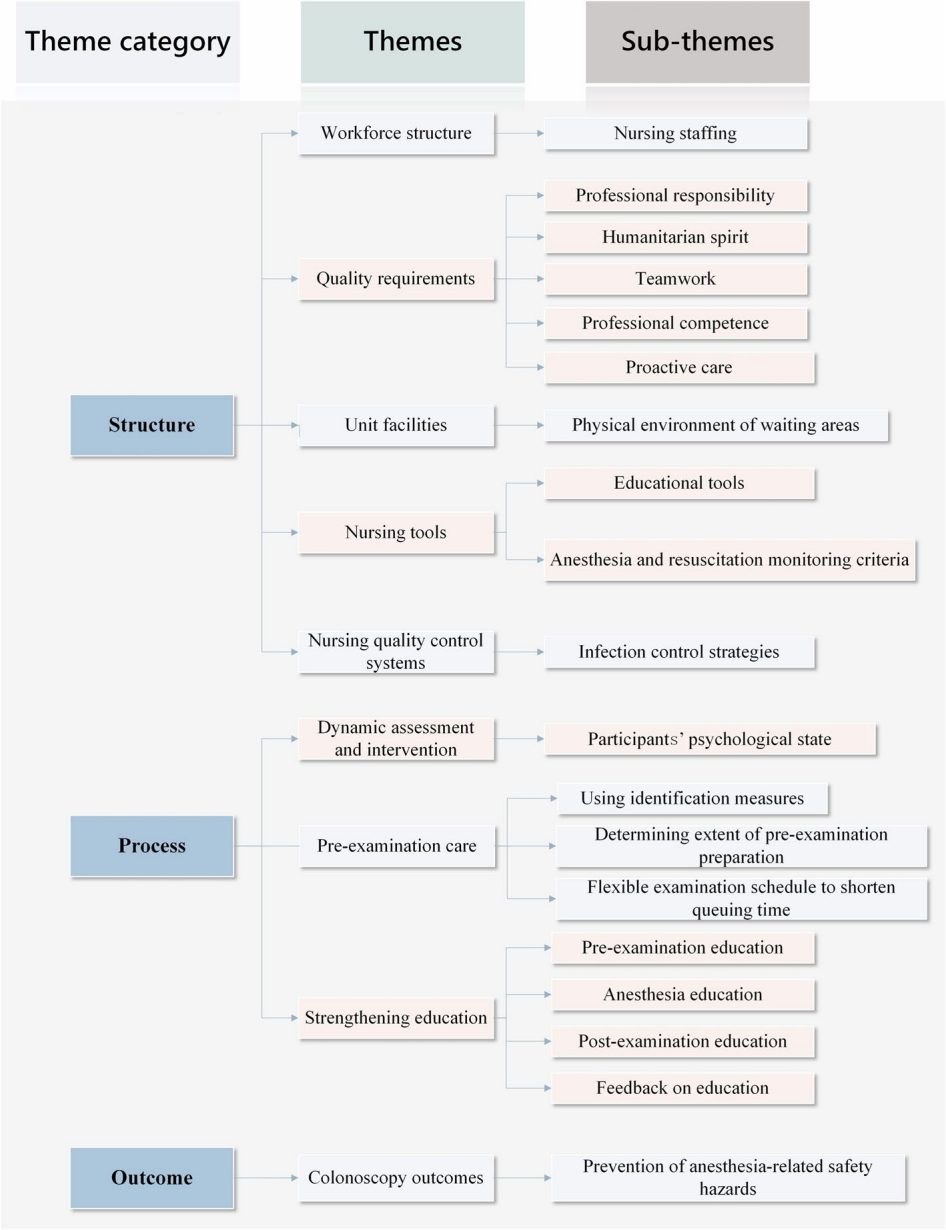
Study themes guided by the Donabedian theory

Eleven colonoscopy participants and seven endoscopy unit nurses were included. Table 2 presents the demographic characteristics of the interviewees. From the interview data, we extracted nine themes based on the structure-process-outcome model: workforce structure, quality requirements, unit facilities, nursing tools, nursing quality control systems, dynamic assessment and intervention, pre-examination care, strengthening education, and colonoscopy outcomes, and 19 subthemes. The themes, subthemes, and exemplar quotes of the interviews are presented in Fig. 1 and Supplementary Table 1, respectively. Participants were represented by P1-P11 and nurses by N1-N7.

**Table 2:** Demographic characteristics of interviewees

Variable	Frequency (%)	Variable	Frequency (%)
Participants’ characteristics (*P* = 11)		Nurses’ characteristics (*N* = 7)	
**Age (years)**		**Age (years)**	
≤ 30	1 (9.1)	26 ~ 35	2 (28.6)
31 ~ 40	4 (36.4)	36 ~ 45	3 (42.9)
41 ~ 50	3 (27.3)	46 ~ 55	2 (28.6)
> 50	3 (27.3)	**Gender**	
**Gender**		Men	1 (14.3)
Men	6 (54.5)	Women	6 (85.7)
Women	5 (45.5)	**Education**	
**Occupational status**		Below bachelor's degree	3 (42.9)
Employed	10 (90.9)	Bachelor degree	3 (42.9)
Unemployed	1 (9.1)	Master degree	1 (14.3)
**Marital status**		**Years in profession (years)**	
Single/divorced	1 (60.2)	6 ~ 10	1 (14.3)
Married	10 (30.6)	11 ~ 15	4 (57.1)
**Education**		> 15	2 (28.6)
Below bachelor's degree	3 (27.3)		
Bachelor degree or above	8 (72.7)		
**Examination reason**			
With symptoms	3 (27.3)		
No symptoms	8 (72.7)		
**Examination results**			
All is well	4 (36.4)		
Intestinal adenoma/ulcer	7 (63.6)

### Workforce structure

#### Nursing staffing

The physical strength of nurses is increasing at an accelerated rate due to the high-intensity, high-stress working environment in the endoscopy unit. Unreasonable staffing may exacerbate this situation and increase the incidence of adverse nursing events. Therefore, nurses (4/7) proposed staffing requirements to provide safer and more effective care for colonoscopy participants.

“It might be better to restructure staffing in the future. Having a dedicated position for everyone will ensure a smooth workflow. In fact, the higher the workload, the higher the risk of making mistakes.” (N4).

### Quality requirements

#### Professional responsibility

One participant (1/11) mentioned that the professional responsibility of nurses is a noble and valuable quality necessary for nurses in their work.

“My understanding is that nurses should carry out their duties; responsibility is both noble and mundane and needs to be consciously obeyed by the nurses.” (P6)

#### Humanitarian spirit

The experiences of nurses (1/7) and colonoscopy participants (4/11) indicated that sincerity and patience during the nursing process could reduce their anxiety. The participants appreciated the patience and kindness of the nurses they felt during care and considered a good attitude toward caring as one of the criteria for their choice of medical institution.

“I think the attitude of nurses is quite essential, because I feel more relaxed when nurses have a better attitude.” (P1)

#### Teamwork

Colonoscopy requires close cooperation and effort from a team. The nurses (2/7) were immersed in a congenial teamwork environment, and they described how colleagues helping each other in the workplace could improve overall productivity.

“Work becomes efficient because of timely cooperation.” (N1)

#### Professional competence

Both colonoscopy participants (3/11) and nurses (3/7) cited the need for professional competence during the interviews. Prior to deciding on a colonoscopy site, participants determined their level of care by asking friends or relatives who had experience with the exam, searching online, and consulting with a medical professional, which increased their sense of security.

“Professionalism of the medical team comes first in my list of importance.” (P6)

#### Proactive care

Although participants had varied opinions about the care they received, it was clear that all participants expected nurses to be more proactive during their care and provide additional help beyond what they needed. Mechanical care that did not meet their expectations caused them to feel frustration.

“I had no idea about what to do afterwards, and no one told me. I felt a little confused about the process. The nurse just called out my name and told me to change and keep lying down. I would have shared my queries with the nurse, but I didn't bother when I noticed that they were busy.” (P6)

One nurse (1/7) also believed that providing attentive and proactive care to the participants would increase their satisfaction.

“I believe it might be better if the nurses explained everything before being asked for clarifications by the participants.” (N1)

### Unit facilities

#### Physical environment of waiting areas.

The structural setup and facilities of the endoscopy room play an important role in calming the examiners. One nurse (1/7) emphasized that a suitable environment could alleviate participants’ and staff’s anxiety and avoid conflict to some extent.

“I think it's important for everyone to have a nice environment, whether it's the staff or the patients. It makes a person comfortable and is less irritating.” (N1)

### Nursing tools

#### Educational tools

Two nurses (2/7) emphasized the importance of tool use and demonstrated that precautionary instructions were distributed to the participants before and after colonoscopy. However, participants' responses were variable, with one participant (1/11) feeling that he received practical and reliable information; they were able to review it themselves to prevent ambiguity and reduce the burden on nurses. Some participants (3/11) said that they were completely unfamiliar with the precautions they were provided with, and the time lapse between preaching and implementation exacerbated the forgetfulness of knowledge. Therefore, participants would like to have more detailed guidelines to guide them. In addition, participants were looking forward to an educational approach using the Internet.

“An appointment form with various notes is necessary.” (N2)

#### Anesthesia and resuscitation monitoring criteria

The majority of participants chose painless colonoscopy, which adds a degree of challenge to colonoscopy. The nurses (2/7) showed that the procedure was more demanding, which made them spend a lot of effort monitoring the vital signs and mental status of the participants after anesthesia to ensure their safety.

“As soon as a participant finishes medical examination, we will record it on the resuscitation record sheet, including the participant's blood oxygen, heart rate level, as well as the participant's departure time.” (N1)

### Nursing quality control system

#### Infection control strategies

Strict aseptic practices provided safety to colonoscopy participants. In particular, colonoscopy cleaning and disinfection were suggested by most nurses (3/7) as an important part of facilitating colonoscopy infection control efforts.

“Our infection control requirements are strict and rigorous as cleaning and disinfection are important for patient safety.” (N6)

### Dynamic assessment and intervention

#### Participants’ psychological state

During colonoscopy, participants felt worried and anxious when faced with an unknown environment and operation, which was especially evident among participants who were attending the examination for the first time. However, such a situation was not detected and resolved by the nurses, leaving the participants ambivalent and nervous during the examination.

“I feel embarrassed because wearing the given pants will reveal my buttocks if I'm not careful as the colonoscope has to go in through the anus.” (P5)

### Pre-examination care

#### Using identification measures

Nurses need feedback from participants regarding their names when confirming their identities to ensure the accuracy and safety of care. One participant (1/11) praised the nurse's practice of checking his or her identity. The same topic was mentioned by one nurse (1/7).

“It is necessary to check a patient's name and other information because calling a patient directly and asking for the name are two different things, and the latter prevents confusion about the patient's identity. Thus, we must ask the patient's name rhetorically in clinical practice.” (N1)

#### Determining extent of pre-examination preparation

It is essential to ask participants about the use of bowel-cleansing medication, diet, and bowel movements before the examination to help avoid repeat examinations in a short time due to unqualified bowel preparation. It also helps to protect the participant's safety from anesthesia and avoid disputes due to invalid tests. Although the nurse is often frustrated by their lack of cooperation, she insists on asking them about their bowel preparation.

“It's hard to figure out whether the patient's bowel is completely prepared. Thus, we have to ask the following questions: ‘What time did you finish taking the morning medicine? Did you have watery stools after taking the medicine? Did you drink and eat again after taking the medicine?’” (N2)

#### Flexible examination schedule to shorten queuing time

During the interviews, the nurses (2/11) indicated that they considered reducing queuing time for the participants. Some participants (2/11) also described their own experiences of impatience due to long waiting times before the examination, which made them abandon their original colonoscopy site. On the contrary, colonoscopy with an appropriate waiting time would increase participant satisfaction. Another participant (1/11) was surprised by the short queue because he was mentally prepared for a long wait.

“I took note of the time. I went in at 3:10 p.m., and the examination took a total of 45 minutes. It didn't seem to take as long as I thought it would.” (P2)

### Strengthening education

#### Pre-examination education

For the participants, quality bowel cleansing could not be achieved if they did not receive detailed pretest education in the hospital. Participants (5/11) described uncertainty about their bowel-cleansing methods and outcomes. And they were also unable to counter the adverse effects of the medication, which led to a bad medication experience.

“This could have occurred because of a poor response to the medication. I am uncertain regarding the frequency and extent of defecation that is needed prior to the colonoscopy.” (P1)

“I woke up at 4 am and remained awake until the next morning because I had to defecate. The medicine was so unpleasant that I vomited. Moreover, I had a bloating sensation because of drinking an excessive amount of water.” (P5)

#### Anesthesia education

Participants often think of the adverse effects of anesthesia when referring to intravenous anesthesia, which creates a crisis of confidence in anesthesia. Some participants in the gastrointestinal examination were not told what liquid they drank before the procedure and could only guess that it was narcotic according to physiological sensations.

“I wasn't quite sure what the effect of the ingested throat anesthetic would be; I thought it would make me unconscious like a general anesthetic, but that didn't actually seem to be the case.” (P4)

#### Post-examination education

Detailed post-examination precautions explained by the nurses helped the recovery of participants. Some participants (1/11) felt that they were given adequate explanations of the post-examination precautions. However, some (2/11) had difficulty understanding and mastering self-management, and they reported that the nurses did not provide useful information.

“Yes, I was confused and checked the related information yesterday because I could not remember the nurse’s instruction regarding the time of eating and drinking.” (P6)

#### Feedback on education

Nursing education should be interactive, as suggested by both nurses (2/7) and participants (1/11). Getting feedback from participants through interactions and making timely corrections can ensure the accuracy and effectiveness of education.

“I think it is important that the education is interactive. The nurses should confirm our understanding of the instructions. I think it is probably better for the nurses to repeat the instructions for our better understanding.” (P7).

### Colonoscopy Outcomes

#### Prevention of anesthesia-related safety hazards.

During the interviews, most nurses (4/7) made special comments regarding the safety of the participants. Special attention should be paid to the mental state of the examinee after resuscitation with anesthesia. For nursing staff, post-resuscitation conditions are unknown and unpredictable; therefore, they need to be prepared.

“In these patients, falls are considered serious and dangerous nursing accidents.” (N4)

## Discussion

This study aimed to gain insight into nursing-related intervention valued by colonoscopy participants, and nurses' attitudes toward optimize the quality of care. These results provide a help for developing quality indicators for colonoscopy care.

From the level of nursing structure, low nurse staffing levels have a negative impact on quality of care [22, 23]. Therefore, nursing managers should focus on rationalizing the staffing of nursing units in endoscopy units. Although recruiting more nursing staff is the most immediate solution, the worldwide shortage of nursing labor has increased during the COVID-19 pandemic. This suggests that nursing managers may also need to consider other strategies, such as improving the overall resilience of the nursing workforce to make more of a difference with the limited available human resources [24]. The proactive and caring behavior of the nurses toward the participants were described as necessary factors to improve their experience. And nurses who focus on their communication skills and maintain an appropriate pace and tone of voice can convey patience, attention, and support to participants [25]. In addition, a good waiting environment and suitable waiting time were both priorities when participants chose a medical institution for colonoscopy. In conclusion, all these details should be attended to by the nursing workforce to improve participant satisfaction and the overall competitiveness of the endoscopy unit. Medication and diet control were important aspects of colonoscopy. However, the majority of participants were unable to absorb sufficient and effective information from existing education. It can be observed that the participants had uncertain experiences about self-management methods as well as the effects before and after their examinations. In addition, we noted that participants were used to searching the Internet for information about colonoscopy, but the accuracy of unofficially published colonoscopy-related information on the Internet remains to be verified because there is no guarantee that all posters on the Internet acquired specialized training in colonoscopy, and it is difficult to guarantee the credibility of the posters [26]. The result is that participants who lack professional knowledge may be misled when searching the Internet because of their inability to screen the authenticity of such knowledge. Thus, a combination of these two points suggests that nurses need to provide educational tools that can be easily understood by most participants. These tools should be aesthetically appealing as well as practical and should be disseminated through offline instruction and the Internet. Therefore, intensive education with the development of such clear and easy-to-understand educational tools as part of quality of care assessment may contribute to assisting participants' understanding of colonoscopy-related knowledge. In addition to aid, nurses should be mindful of how to set standards to standardize nursing practices to safeguard the health outcomes of participants. For example, nurses agree that hospital and endoscopy units impose strict requirements in terms of sedation and infection control, and they believe that the continued safety of colonoscopy cannot be achieved without quality control at every step of the process.

The process of care was concerned by the nurses as well as the participants. It is worthwhile to investigate deeply why some of the participants expressed a range of negative emotions including doubts, worries, nervousness, and fears. Negative emotions may have a direct impact on screening behavior and compliance with screening [27]. This significant factor influencing the decision-making of participants should be considered by nurses, and a dynamic assessment of a range of psychological states should be conducted to discover these emotions early so that we can prepare to respond to them in a timely manner. Furthermore, although the participants in the study passed the examination successfully, the process was cumbersome. The most common difficulties were the inability to fully master the medication administration and technique, causing a tendency to suffer from bloating, nausea, and vomiting, which was a major factor contributing to the participants' lack of confidence in bowel cleansing. To this end, nursing managers should organize nurses to collect the practical needs of colonoscopy participants in a timely manner and provide them with targeted discomfort-relieving reinforcement instructions, including verbal and written instructions, to help them achieve adequate bowel cleansing. Feedback is an important part of ensuring learning goals, and learners receive more information when they provide feedback to the instructor [28]. Accordingly, we encourage nurses to develop the concept of nurse-patient interaction and focus on educational feedback, which will help enhance the overall quality of nursing staff and improve the nurse-patient relationship.

Nursing outcome is the embodiment of the quality of nursing care. Despite the rate of interval CRC being the ultimate gold standard measure of colonoscopy quality [6], nurses’ views on outcome indicators still revolved around ensuring the safety of participants in our study, and they had extensive experience in preventing anesthesia-related safety hazards. Participants did not specifically comment on examination outcomes, possibly because no adverse outcomes occurred among the participants included in this study, so participants were more inclined to focus on their own experiences instead of adverse events that did not occur. However, the incidence of all adverse outcomes increases as the number of participants increases [2, 29]. It is valuable to create more quality indicators to evaluate care outcomes so that caregivers can clearly identify and control adverse events.

### Limitations

Although data analysis has reached saturation, this study did not include samples from different levels of hospitals, resulting in a single-source sample that may not be enough to cover a broader range of themes. Moreover, we only included the views of the participants and nursing staff and did not involve other healthcare professionals, which may not be representative of the views of the entire healthcare community.

## Conclusion

Our study provided insight into the caring requirements of colonoscopy participants and nurses and clarified the shortcomings and room for improvement of care practices. This study suggests that more nursing managers should pay attention to this area in the future and collect the needs and suggestions of the colonoscopy-related population to guide the quality management of colonoscopy care, which will contribute to better colonoscopy care and have a significant impact on the increase in satisfaction of the participant.

## Supplementary Information

**Additional file 1.** CONSOLIDATED CRITERIA FOR REPORTING QUALITATIVE RESEARCH (COREQ): 32-item checklist.

**Additional file 2: Supplementary table 1.** Complete table of themes, Subthemes and example quotes.

## Data Availability

The datasets generated and analysed during the current study (interview transcripts and recording material) are not publicly available due to participant confidentiality considerations. Aggregate data are available from the corresponding author upon reasonable request.

## Abbreviations

CRC: Colorectal cancer
AMR: Adenoma miss rate
PCN: Patient-centered care
COREQ: Consolidated criteria for reporting qualitative research
