# Supplementary material for: Quality indicators of colonoscopy care: a qualitative study from the perspectives of colonoscopy participants and nurses
Source: BMC Health Serv Res. 2022 Aug 19;22:1064. doi: 10.1186/s12913-022-08466-5 (PMC9390113; doi:10.1186/s12913-022-08466-5)
Supplement: Supplementary file 1 — Additional file 1. CONSOLIDATED CRITERIA FOR REPORTING QUALITATIVE RESEARCH (COREQ): 32-item checklist. [file 12913_2022_8466_MOESM1_ESM.docx]

**CONSOLIDATED CRITERIA FOR REPORTING QUALITATIVE RESEARCH (COREQ): 32-item checklist**

| **No.** | **Item** | **Guide Questions/Description** | **Reported on Page No.** |
| --- | --- | --- | --- |
| **Domain 1: Research team and reflexivity**  Personal characteristics | | | |
| 1 | Interviewer/facilitator | Which author/s conducted the interview or focus group? | The first author.  Page 5/ Authors’ contributions |
| 2 | Credentials | What were the researcher’s credentials? E.g. PhD, MD | BSN |
| 3 | Occupation | What was their occupation at the time of the study? | Page 5 |
| 4 | Gender | Was the researcher male or female? | Page 5 |
| 5 | Experience and training | What experience or training did the researcher have? | Page 5 |
| Relationship with participantss | | | |
| 6 | Relationship established | Was a relationship established prior to study commencement? | Page 5 |
| 7 | Participant knowledge of the interviewer | What did the participants know about the researcher? e.g., personal goals, reasons for doing the research | Page 5 |
| 8 | Interviewer characteristics | What characteristics were reported about the inter viewer/facilitator? e.g., Bias, assumptions, reasons, and interests in the research topic | Page 5 |
| **Domain 2: Study design**  Theoretical framework | | | |
| 9 | Methodological orientation and Theory | What methodological orientation was stated to underpin the study? e.g., grounded theory, discourse analysis, ethnography, phenomenology, content analysis | Page 4 |
| Participant selection | | | |
| 10 | Sampling | How were participants selected? e.g., purposive, convenience, consecutive, snowball | Page 4 |
| 11 | Method of approach | How were participants approached? e.g., face-to-face, telephone, mail, email | Page 4 |
| 12 | Sample size | How many participants were in the study? | Page 6 |
| 13 | Non-participation | How many people refused to participate or dropped out? Reasons? | No drop out. |
| Setting | | | |
| 14 | Setting of data collection | Where was the data collected? e.g., home, clinic, workplace | Page 4 |
| 15 | Presence of nonparticipants | Was anyone else present besides the participants and researchers? | Page 4 |
| 16 | Description of sample | What are the important characteristics of the sample? e.g., demographic data, date | Table 2 |
| Data collection | | | |
| 17 | Interview guide | Were questions, prompts, guides provided by the authors? Was it pilot tested? | Page 5 and Table 1 |
| 18 | Repeat interviews | Were repeat interviews carried out? If yes, how many? | No. |
| 19 | Audio/visual recording | Did the research use audio or visual recording to collect the data? | Page 5 to 6 |
| 20 | Field notes | Were field notes made during and/or after the interview or focus group? | Page 5 |
| 21 | Duration | What was the duration of the inter views or focus group? | Page 5 |
| 22 | Data saturation | Was data saturation discussed? | Page 4 |
| 23 | Transcripts returned | Were transcripts returned to participants for comment and/or correction? | No |
| **Domain 3: analysis and findings**  Data analysis | | | |
| 24 | Number of data coders | How many data coders coded the data? | Page 5 to 6 |
| 25 | Description of the coding tree | Did authors provide a description of the coding tree? | No |
| 26 | Derivation of themes | Were themes identified in advance or derived from the data? | Page 5 to 6 |
| 27 | Software | What software, if applicable, was used to manage the data? | Page 5 |
| 28 | Participant checking | Did participants provide feedback on the findings? | No |
| Reporting | |  |  |
| 29 | Quotations presented | Were participant quotations presented to illustrate the themes/findings? Was each quotation identified? e.g., participant number | Page 6 to 15 |
| 30 | Data and findings consistent | Was there consistency between the data presented and the findings? | Page 6 to 15 and Supplementary Table 1 |
| 31 | Clarity of major themes | Were major themes clearly presented in the findings? | Page 6 to 15 and Table 3 |
| 32 | Clarity of minor themes | Is there a description of diverse cases or discussion of minor themes? | Page 6 to 15 |

Developed from: Tong, A., Sainsbury, P., & Craig, J. (2007). Consolidated criteria for reporting qualitative research (COREQ): a 32-item checklist for interviews and focus groups. International Journal for Quality in Health Care, 19(6), 349-357. doi: 10.1093/intqhc/mzm042
