# Supplementary material for: Quality indicators of colonoscopy care: a qualitative study from the perspectives of colonoscopy participants and nurses
Source: BMC Health Serv Res. 2022 Aug 19;22:1064. doi: 10.1186/s12913-022-08466-5 (PMC9390113; doi:10.1186/s12913-022-08466-5)
Supplement: Supplementary file 2 — Additional file 2: Supplementary table 1. Complete table of themes, Subthemes and example quotes. [file 12913_2022_8466_MOESM2_ESM.docx]

| **Supplementary Table 1. Complete table of themes, Subthemes and example quotes.** | | | |
| --- | --- | --- | --- |
| Theme category | Themes | Subthemes | Quotes |
| Structure | Workforce structure | Nursing staffing | Adequate human resources can increase the success rate of gastroscopy. (N2) |
|  |  |  | Our operating efficiency was pushed beyond its maximum capacity. However, it may breakdown if the inflow of patients continue to increase further because we are understaffed. It might be better to restructure staffing in the future. Having a dedicated position for everyone will ensure a smooth workflow. In fact, the higher the workload, the higher the risk of making mistakes. (N4) |
|  |  |  | Our regular job does not only involve cleaning and disinfecting colonoscopes either; we often have to multitask. (N5) |
|  |  |  | I think a well-established endoscopy center must be well-staffed. (N7) |
|  | Quality requirements | Professional responsibility | My understanding is that nurses should carry out their duties; responsibility is both noble and mundane and needs to be consciously obeyed by the nurses. (P6) |
|  |  | Humanitarian spirit | I think the attitude of nurses is quite essential, because I feel more relaxed when nurses have a better attitude. (P1) |
|  |  |  | We mainly need nurses to explain the relevant details to us patiently and clearly. (P2) |
|  |  |  | The nurses were very good at patiently answering all my questions. What impressed me was that I was guided and accompanied by a nurse upon arriving at each unfamiliar consultation room. (P7) |
|  |  |  | I've seen some patient nurses before, but the ones here are different. (P9) |
|  |  |  | When I sincerely explained to the patients that they needed to be patient in the queue, they understood and were reasonable. (N3) |
|  |  | Teamwork | Work becomes efficient because of timely cooperation. (N1) |
|  |  |  | It is beneficial to help each other as a team. (N2) |
|  |  | Professional competence | I chose this hospital because I was assured by the skills of the medical team here. (P3) |
|  |  |  | Professionalism of the medical team comes first in my list of importance. (P6) |
|  |  |  | I believe that successful completion of colonoscopy is dependent on a high level of technical skills and extensive operational experience. (P10) |
|  |  |  | The nurse on duty must pass the induction training and evaluation by an instructor to confirm whether the nurse can operate independently. (N2) |
|  |  |  | The nurse must be professional, qualified, and specialized.（N6） |
|  |  |  | In addition to passing the induction training, nurses are required to visit specialized endoscopy units for operational training to improve knowledge and skills. (N7) |
|  |  | Proactive care | The nurse would first talk to me about the next steps and answer all my questions very clearly. (P1) |
|  |  |  | I felt comfortable because the nurse would initiate a conversation about something to relax me. (P3) |
|  |  |  | As soon as I arrived, the nurse clearly explained each procedure to me. (P4) |
|  |  |  | I had no idea about what to do afterwards, and no one told me. I felt a little confused about the process. The nurse just called out my name and told me to change and keep lying down. I would have shared my queries with the nurse, but I didn't bother when I noticed that they were busy. (P6) |
|  |  |  | I was reassured because the nurse clarified all the operation preparations, even the change of pants. (P8) |
|  |  |  | I believe it might be better if the nurses explained everything before being asked for clarifications by the participants. (N1) |
|  | Unit facilities | Physical environment of waiting areas | The environment is good, and the facilities are complete; so all in all, I’m satisfied.（P2) |
|  |  |  | The waiting area and restrooms here are better than those in the other units. They are clean and tidy. (P7) |
|  |  |  | The environment is nice and quiet. I feel soothed and relieved. (P11) |
|  |  |  | I think it's important for everyone to have a nice environment, whether it's the staff or the patients. It makes a person comfortable and is less irritating. (N1) |
|  | Nursing tools | Educational tools | The nurse was busy while talking about the precautions. Thus, the precautions weren’t particularly clear. I also didn't pay attention to the details, probably because it was a week apart. I suggested the nurse to send me the details about the food precautions via email or WeChat for convenience. I believe it will be beneficial to clarify what events were a week apart. As I wasn’t sure of the specific defecation requirements, I did some googling. (P1) |
|  |  |  | Nurses sharing with us the whole process in advance will reduce our confusion and make the nurse's job comparatively easier. (P4) |
|  |  |  | If the nurse shares a detailed follow-up note, I can check it again whenever I feel unsure. However, if the nurse only verbally explains it, I might have to come back to the hospital to ask her for clarification, which is very troublesome. Thus, a detailed note is crucial. I look for information online if I don’t understand something. Moreover, I use Red or Baidu to search. (P6) |
|  |  |  | Clear information sheets should be available. I just heard a guy say, 'I forgot.' Thus, we may not really be able to remember the briefly listed information shared by nurses, and having an information sheet would make us feel more confident. (P7) |
|  |  |  | I search the internet, for example, to find an authoritative hospital for gastroscopy. (P9) |
|  |  |  | I found a lot of information about a colonoscopist on TikTok. (P10) |
|  |  |  | An appointment form with various notes is necessary. (N2) |
|  |  |  | Written instructions are certainly convenient and necessary, as participants may forget relevant details. (N3) |
|  |  | Anesthesia and resuscitation monitoring criteria | As soon as a participant finishes medical examination, we will record it on the resuscitation record sheet, including the participant's blood oxygen, heart rate level, as well as the participant's departure time. (N1) |
|  |  |  | The resuscitation nurse must be professional who assesses whether the patient is ready to leave the resuscitation room after awakening based on the criteria. (N6) |
|  | Nursing quality control systems | Infection control strategies | Cleaning and disinfecting a colonoscope must be done carefully as the hospital administration inspects infection control measures every quarter. (N2) |
|  |  |  | We must ensure that the colonoscope is cleaned and disinfected. (N5) |
|  |  |  | Our infection control requirements are strict and rigorous as cleaning and disinfection are important for patient safety. (N6) |
| Process | Dynamic assessment and intervention | Participants’ psychological state | I'm still a little worried because this is a general anesthesia test. (P1) |
|  |  |  | I feel embarrassed because wearing the given pants will reveal my buttocks if I'm not careful as the colonoscope has to go in through the anus. (P5) |
|  |  |  | I was a little nervous at first because this was my first colonoscopy, and I was concerned about additional damage to my digestive tract. (P7) |
|  |  |  | I was nervous because it was my first time undergoing general anesthesia. (P9) |
|  |  |  | The main concern with colonoscopy is of additional damage to the intestines and stomach. (P10) |
|  | Pre-examination care | Using identification measures | The nurse asked for my name, which I thought was important. (P8) |
|  |  |  | It is necessary to check a patient's name and other information because calling a patient directly and asking for the name are two different things, and the latter prevents confusion about the patient's identity. Thus, we must ask the patient's name rhetorically in clinical practice. (N1) |
|  |  | Determining extent of pre-examination preparation | It's hard to figure out whether the patient's bowel is completely prepared. Thus, we have to ask the following questions: ‘What time did you finish taking the morning medicine? Did you have watery stools after taking the medicine? Did you drink and eat again after taking the medicine?’ (N2) |
|  |  | Flexible examination schedule to shorten queuing time | My experience at the previous clinic was unpleasant. Many people were waiting inside; I waited for a long time, but the clinic remained crowded. (P1) |
|  |  |  | I took note of the time. I went in at 3:10 p.m., and the examination took a total of 45 minutes. It didn't seem to take as long as I thought it would. (P2) |
|  |  |  | There are differences among clinics. The clinic here is less crowded because of few people and short waiting lines. (P7) |
|  |  |  | The following is written on all our appointment sheets: Please leave half a morning free to wait patiently. I'm specifically going to circle this line. (N2) |
|  |  |  | For example, if we schedule all patients for examination in the morning and those for treatment in the afternoon, I think the success rate and patient satisfaction will improve. (N4) |
|  | Strengthening education | Pre-examination education | This could have occurred because of a poor response to the medication. I am uncertain regarding the frequency and extent of defecation that is needed prior to the colonoscopy. I took the laxative thrice, but the taste was slightly unpleasant. There was minimal vomiting when I ingested the laxative hurriedly. (P1) |
|  |  |  | We are uncertain regarding the procedure of ingesting the cleansing medication. We may have missed the cue regarding the timepoint when drinking water was allowed as the nurse spoke quickly. (P7) |
|  |  |  | As the instructions indicated that movement can accelerate bowel movement, I followed it. (P2) |
|  |  |  | I woke up at 4 am and remained awake until the next morning because I had to defecate. The medicine was so unpleasant that I vomited. Moreover, I had a bloating sensation because of drinking an excessive amount of water. (P5) |
|  |  |  | The medicine was difficult to ingest and resulted in slight discomfort. I was unable to drink the second pot of medicine without difficulty, and it resulted in vomiting. (P10) |
|  |  | Anesthesia education | I believe that general anesthesia has some side effects. (P3) |
|  |  |  | I wasn't quite sure what the effect of the ingested throat anesthetic would be; I thought it would make me unconscious like a general anesthetic, but that didn't actually seem to be the case. (P4) |
|  |  |  | I am unaware of the medication, but it could have been an anesthetic agent. (P6) |
|  |  | Post-examination education | I feel like I can until I am satisfied after the checkup. (P5) |
|  |  |  | Yes, I was confused and checked the related information yesterday because I could not remember the nurse’s instruction regarding the time of eating and drinking. (P6) |
|  |  |  | Since we were concerned about the diet, the nurse explained all instructions clearly, including a list of semi-liquid and liquid foot items.(P8) |
|  |  | Feedback on education | I think it is important that the education is interactive. The nurses should confirm our understanding of the instructions. I think it is probably better for the nurses to repeat the instructions for our better understanding. (P7) |
|  |  |  | I think that the instructions should be repeated for the patients’ benefit. If patient is able to repeat my instructions, it is clear that have understood them. Otherwise, I repeat the instructions until the patient can understand them. (N1) |
|  |  |  | In particular, precise and effective patient education is important at all times. (N6) |
| Outcome | Colonoscopy outcomes | Prevention of anesthesia-related safety hazards | The anesthetized patient needs to sit up and rest immediately after awakening. Movement should be restricted until the patient is comfortable enough to stand up. I also instruct the family members to accompany the patient when changing to ensure safety. (N1) |
|  |  |  | A patient who has just recovered from anesthesia must be well taken care of. It is important to protect the patient from falls in this state. (N2) |
|  |  |  | After recovering from anesthesia, the patient will be prone to accidents. Therefore, we instruct patients who are scheduled for anesthesia to bring their family members to the hospital. (N3) |
|  |  |  | In these patients, falls are considered serious and dangerous nursing accidents. (N4) |
